# Cryopreservation of Animals and Cryonics: Current Technical Progress, Difficulties and Possible Research Directions

**DOI:** 10.3389/fvets.2022.877163

**Published:** 2022-06-09

**Authors:** Marlene Davis Ekpo, George Frimpong Boafo, Suleiman Shafiu Gambo, Yuying Hu, Xiangjian Liu, Jingxian Xie, Songwen Tan

**Affiliations:** ^1^Xiangya School of Pharmaceutical Sciences, Central South University, Changsha, China; ^2^Department of Orthopedic Surgery, The Second Xiangya Hospital of Central South University, Central South University, Changsha, China

**Keywords:** cryonics, cryostasis, cryoprotectants, medical cryopreservation, cryopatient

## Abstract

The basis of cryonics or medical cryopreservation is to safely store a legally dead subject until a time in the future when technology and medicine will permit reanimation after eliminating the disease or cause of death. Death has been debunked as an event occurring after cardiac arrest to a process where interjecting its progression can allow for reversal when feasible. Cryonics technology artificially halts further damages and injury by restoring respiration and blood circulation, and rapidly reducing temperature. The body can then be preserved at this extremely low temperature until the need for reanimation. Presently, the area has attracted numerous scientific contributions and advancement but the practice is still flooded with challenges. This paper presents the current progression in cryonics research. We also discuss obstacles to success in the field, and identify the possible solutions and future research directions.

## Introduction

In an era of limitless scientific possibilities, humans are continually pushing boundaries to bring to reality the seemingly unimaginable. A perfect example of such innovation is the preservation of whole animals and humans at cryogenic temperature with the intention of restoring good health and resurrection in the future (1). Cryopreserving animals especially endangered species can be applicable to preventing extinction. Cryonicists hold the opinion that the pronouncement of legal death does not infer an irreversible event and that the medical technology required to initiate this reverse is presently unlocked (2).

Some proven fundamental principles of cryopreservation and cryobiology govern the practice of cryonics (2). These principles include: (A) Hypothermia can reduce or pause metabolism and other biochemical reactions while offering protection against ischemic injury. This is a natural occurrence for the northern wood frog (*Rana sylvatica*), which adapts to subzero temperatures (−3 to −6°C) for prolonged durations by transitioning into a semi-frozen phase devoid of cardiac function (3). Clinically, cryopreservation technology is applied extensively to extend the survival time of different cells, tissues and organs which would naturally lose viability if left unpreserved. Also, several research studies are presently being conducted to optimize cryopreservation of different materials (4, 5). (B) Cryoprotectants otherwise called cryoprotective agents (CPAs) can reduce or inhibit ice formation and nucleation (6). The effective application of cryoprotectants has been in existence as far back as the 1960's when glycerol was used by a Japanese scientist for the cryopreservation of cat brain at −20°C. Upon thawing after 45 days and electroencephalogram examinations, the brain displayed normal activity (7). Cryopreservation is performed either through programmable slow freezing, vitrification or low-CPA vitrification (ultra-rapid cooling) with vitrification being is the preferred technique in cryonics because it prevents/reduces the formation of damaging ice crystals within the cryopreserved subject (8).

**Figure 1:**
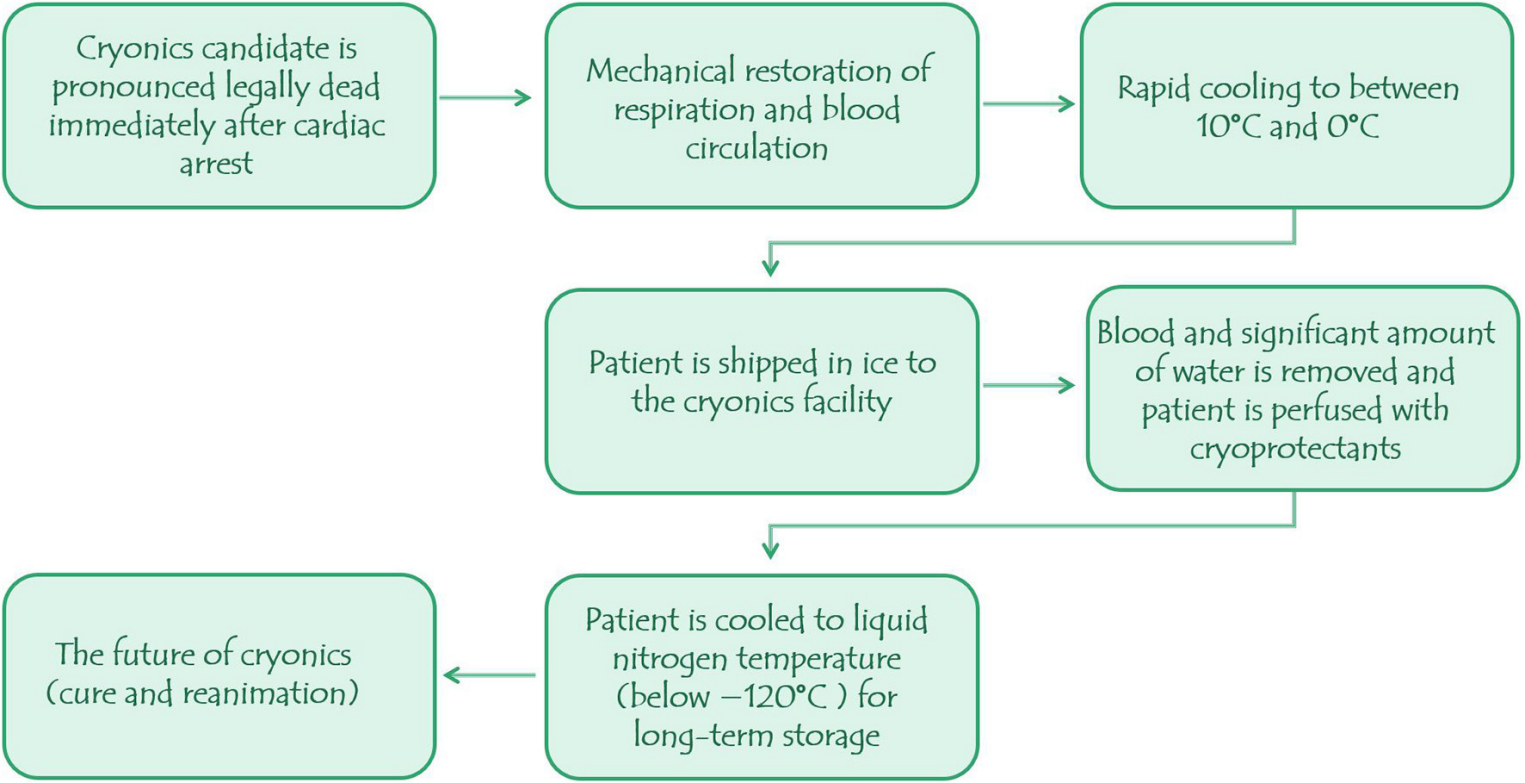
The procedure in cryonics.

To carry out cryonics presently, the candidate must be declared legally dead and consent must have been obtained prior because the procedure is yet to obtain medical recognition and approval (9). As shown in Figure 1, the cryopreservation procedure should commence ideally within 1–2 min post-mortem.

The initial cooling of the subject to below 10°C is a crucial step required to cease biochemical and metabolic processes. It is usually carried out in a bath containing ice water and the cooling transfer is mainly controlled by convection, fluid motion and conduction mechanisms. The assisted blood circulation induced by cardiopulmonary support also facilitates cooling by promoting heat transfer (9). Each step leading to the final placement of the vitrified subject in liquid nitrogen (usually at −196°C) is performed with utmost precision to minimize or prevent further injury to tissues and depending on the cryonics facility, the protocol may vary slightly. For instance, the Alcor cryonics facility; after artificially restoring cardiopulmonary activity infuse intravenous protective medications into the subject in the hope for better outcome (10).

Finally, the patient is preserved in cryostasis in a vacuum-insulated liquid nitrogen vessel awaiting when research answers the question of how to cure and rejuvenate the subject. In this review we contribute toward the way forward in cryonics by presenting current progress and findings in cryonics research like the techniques employed to enhance the neuroprotective effect of hypothermia, restore functioning of the brain and other organs, reduce cryoinjury, extend survival after delayed time to cardiopulmonary resuscitation and minimize perfusion injury. Furthermore, we highlight the challenges encountered and discuss possible research directions.

## Cryonics: From Conception Till Date

Cryonicists share ideologies with anti-aging scientists who propose that aging is a disease treatable with foreseeable medicine after the biomolecular and cellular mechanism of its pathology has been deciphered (11). Human cryostasis was proposed by Robert Ettinger “the father of cryonics” in the 1960s as a means to prevent the brain from deteriorating after legal death (12). The first cryonics patient; Prof. James Bedford remains cryopreserved in the United States of America (USA) (13) and Ettinger became a cryonic patient following his passing in 2011. Some operational cryonics facilities in the world include Alcor Life Extension Foundation (founded 1972) and the Cryonics Institute (founded 1976) both in USA, KrioRus (founded 2005) in Russia and the Shandong Yinfeng Life Science Research Institute (founded 2015) in China.

In 2014, the Alcor Life Extension Foundation claimed to have up to 300 cryonics patients held in their USA facility with more than 1,200 people signing up for the procedure after their demise (2). A recent survey that assessed the knowledge, perception and interest of internet users in the USA on “medical cryopreservation” after death has revealed the propensity of cryonics to gain more recognition (14). A 14-year-old patient who passed away from cancer in 2016 believed cryonics hold the key for her resurrection and cure in the future. Her case was legally upheld and her body is currently cryogenically preserved (15). Nonetheless, while some are still skeptical regarding the success of cryonics, a mean time of 82 years until the revival of cryopreserved bodies has been projected (14).

## Challenges in Cryonics and Cryopreservation of Animals

### Cost

Predictably, cryonics would be expensive because of the large amounts of resources required for prolonged preservation and the scarcity of expertise in the practice. In 2018, affording the procedure was valued at US$28,000 to $200,000 (16) which is significantly high especially for a clinically unproven theory that depends presently on inexistent technology. Notwithstanding, cryonics might be worth the cost should reanimation be achieved in the future.

### Legal Consequences

There is currently no unified global perspective on cryonics. Countries like France and British Columbia have reservations (17) concerning the practice while some others (Germany, Russia and USA) are less stringent (18). Regardless of the technological advancements, cryonics raise some ethical and legal arguments that must be explored. For example, some believe it to be unethical while others think that employing cryonics is justifiable and humane. Other intriguing questions are the legal status of cryopreserved people and if cryothanasia is a feasible option to increase the chances of revival (1).

### Feasibility of Revival

Proving the revival of cryopatients is the most daring obstacle because there are currently no proven interventions that can extend human lifespan (19) talk more of resurrection after legal death. Revival would involve repairing damage caused by freezing (in unvitrified tissues), hypoxia, cryoprotectant toxicity, thermal stress, then cure and possibly tissue regeneration if necessary but valid questions to consider in this regard would be: what does it really entail to be alive? would reversing the cause of death guarantee resurrection or a “healthy corpse”? Presently, research has revealed that different cells, tissues and organs require specifically tailored cryopreservation protocols to promote survival of the cryopreserved material. This requirement would pose a serious challenge to cryonics because the whole body is subjected to the same cryopreservation procedure which may not guarantee revival. Also, a revival protocol would have to be designed for every cryonic patient because people die of innumerable causes.

### Cryopreservation Damage

Increasing the survival of complex tissues and organs remains a difficulty in cryopreservation. Considering the complexity of the human body, recovery from cryoinjury in cryonics would be even more challenging because prolonged vitrification at extremely low temperatures predisposes huge organs to rupture (20, 21). Ice crystals also damage intercellular junctions required for organ functioning (22) and without safe/effective cryoprotectants, cell survival is diminished by dehydration, high salt concentrations and cryoprotectant induced toxicity (23).

### Limited Pro-cryonic Research

Although cryobiology research especially those on cryopreservation of more complex tissues and organs forms the core of cryonics, they cannot count as cryonics studies. According to Alcor, “THE SOCIETY FOR CRIOBIOLOGY has discouraged scientist from doing work that could advance cryonics ….” (24). The predictable outcome of this stance is minor research contributions as seen in Table 1, and slow scientific progress which could be improved upon by encouraging specialization of pro-cryonic scientists and research.

**Table 1:** Summary of original research on cryonics.

**Research aim**	**Methodology**	**Research outcome**	**References**
Investigate the attitude of cryonicists worldwide	Questionnaire survey of 316 people in several cryonics' organizations	70% were members of a cryopreservation body. - “Theory of Cryonic Life Extension” was developed to explains one's interest in cryonics. - The concept of personal identity malleability was proposed.	(2)
To explore the general public attitudes toward cryonics in Germany	Online survey of 1,000 people (age range: from 16 to 69 years old)	47% were aware of cryonics. - 22% could imagine being cryopreserved. - 53% would participate in the latest technological developments.	(25)
Cryopreservation of the human brain.	The brain of a 78-year-old female was perfused with DMSO and glycerol and cryopreserved at 80°C, followed by examination of the integrity of adult neuron marker, doublecortin and synaptophysin in the cryopreserved brain post-thaw.	- Unchanged levels of adult neuron marker in the experimental brain cortex. - Markedly reduced immature doublecortin neurons in the hippocampus in cryoprotected brains. - Synaptophysin markers (hippocampus synaptic network) were intact after cryopreservation	(26)
To measure knowledge, interest and attitudes of internet users toward cryopreservation in USA	Online survey of 1,487 people	75% had previously heard of the topic - 20% expressed interest in signing up - 21% had actively researched the topic. - 6% have chosen to be cryopreserved.	(14)

## Technical Progress and Possible Research Directions in Cryonics

Some view cryonics as fictional. Singh in 2016 states “It appears man is trying to play god, but these attempts are most likely to prove futile. Instead of prolonging human life, it is more important to improve the vitality of health and quality of life throughout the life span of a person” (27). Notwithstanding this notion, researchers are making technical contributions to the field. These studies were mostly not aimed at cryonics but they have contributed substantially and can be considered as research directions in cryonics specialized studies.

### Evaluating the Neuroprotective Effect of Hypothermia

Hypothermia considerably extends tolerance to irreversible ischemic injury (28–30) and many clinical reports, especially pediatrics', exist to support this cerebroprotective property of low temperatures (31). Research with animal models have also confirmed the potential benefits of hypothermia following cardiac arrest (32–34) with most of the neuroprotection attributable to the suppression of neuronal injury (35). Techniques to induce quicker hypothermia are being researched and developed such as cold saline aortic flush tested in pigs (36) and the invention of transnasal high flow dry air (37, 38). Furthermore, candidates like vasopressin (39), dihydrocapsaicin (DHC) (40, 41) and mesenchymal stem cell (MSC) (42) have been evaluated for their neuroprotective enhancing properties.

### Restoring Functioning of the Brain and Other Organs

The closest procedure to cryonics and cryopreservation of whole animals is the cryopreservation of tissues and more complex organs including ovarian tissue (43), bones (44), bone marrow (45), skin grafts (46), pancreatic islet grafts (46), hearts (47), and lungs (48) with some of them being clinically applied during organ transplantation. Several studies are presently geared at improving extending cryopreservation time using suitable techniques and cryoprotectants and concomitantly preserving quality of the cryopreserved material. These studies can serve as insights toward the precise conditions necessary for effective long-term preservation and suggest to cryonics whether to review cryopreservation strategies for increased chances of reanimation.

The brain is highly complex in anatomy and function (49) therefore more difficult to recover after cryopreservation. Anoxia, reperfusion injury, oedema, and metabolic alterations could challenge the integrity of the brain after cryonics exposure and averting them is the aim of some research. Some neuroscientists suggest that memory retention is encoded in the physical structures of the brain, particularly neuropil connectivity and long-term retention of synaptic strengths (50) and possibly within individual neurons through DNA modifications (51). Vrselja et al. developed a cytoprotective and pulsatile-perfusion system that restored brain circulation and cellular functions up to 4 h post-mortem in the pig model (52).

Humans have been subjected to deep hypothermic (16, 24°C) cardiac arrest lasting more than 1 h without gross neurological deficits (53) confirming that for as much as the anatomy is untampered, the brains' electrical activity can be lost and fully recovered (9, 54). Also, the brain has been found intrinsically capable of resisting structural alterations for some time after death. 70–90% of Neurons from autopsied geriatric humans obtained approximately 2.6 h post-mortem remained viable after two (2) weeks *in vitro* (55). Theses research discoveries provide evidence that cryonically preserved subjects might have the chance to recover from ischemia and cryopreservation injury. Neural stem cell therapy (56), digital neural and soma reconstruction (57, 58) using bioabsorbable materials, e.g., polyglycolic acid conduit (59) nanobot cellular repair (60) and neural prosthesis (61) are under investigation as means to improve on restoring brain function. The other less complex organs can be replaced artificially, by tissue engineering (62) or stem cell based regenerative medicine. Regeneration of limbs in salamanders (63) and the use of lab-grown artificial ears (64) are green lights in this direction. Soon, organ replacement by 3D Tissue printing (65) and construction from biodegradable materials, e.g., ligaments with silk collagen scaffold (66) might be put to clinical application.

### Minimizing Cryoinjury and Cryoprotectant Toxicity

A very critical stage in preparing patients for cryostasis is the replacement of body fluids (blood and water) with CPAs which are additives used to minimize ice induced injury to cryopreserved materials and support their post-thaw recovery. Different classes of CPAs exist, each functioning by one or more mechanisms including the induction of thermal hysteresis, ice nucleation and recrystallization inhibition and ice shaping. The most commonly used CPAs in cryobiology are dimethylsulfoxide (DMSO), glycerol, and different polyols like propylene glycol. Vitrification is the preferred cooling method in cryonics as the body is preserved in a stable glassy state (8, 67) but achieving this requires high CPA concentrations which can be damaging and toxic. As the future of cryonics is reanimation, a significant amount of research is currently focused on developing safer and effective CPA options including antifreeze proteins (AFPs) (68), trehalose (69), DP6 (70), nanotechnology engineered cryoprotectants (71), neutral amino acids (6). A major milestone was recorded in 2007 when major cryonics organizations claimed to have successfully vitrified the brain without ice formation (9), still post-thaw recovery not being promised drove the award-winning research of G. Fahy and R. McIntyre in 2015 (72). They infused rabbit brain with glutaraldehyde before vitrification allowing for recovery of an almost perfect brain post-thaw (73).

### Prolonging Survival and Revival After Delayed Time to Cardiopulmonary Resuscitation

Some researchers have suggested that cryothanasia (subjecting a terminally ill patient to cryopreservation prior to legal death) might increase the chances of reanimation (74) but this concept is also hindered by lack of proof and ethical restraints. Experimenting on the concept of cryothanasia might hold the required breakthrough in cryonics but suitable live models have to be used and proven in studies prior to application in humans. In marine and aquatic science, several animals are naturally capable of adapting and surviving hypothermia for prolonged periods during overwintering. This act shares some similarities with cold preserving bodies before death and simulating the natural mechanisms that occur in overwintering animal models may make significant contributions to cryonics.

For instance, ranid frogs are highly sensitive to anoxia and during hypothermia, they maintain normal oxygen uptake and circulation at low oxygen partial pressures by cutaneous gas exchange and diverse physiological changes while energy is supplied by reserves in adipose tissues, liver and muscles (75). This example shows the significance of oxygenation, and justifies the artificial restoration of respiration and blood circulation performed in cryonics. It also reveals that it may be more beneficial to continue the supply of oxygen and energy to the cryopreserved subject throughout the entire cryopreservation process thereby reducing damage induced by hypoxia.

Similarly, Ultsch in 2006 studied different species of turtles undergoing overwintering where it was discovered that most of the marine species respond by migrating to avoid the negative effects of extensive exposure to cold (76). The *Terrapene* turtle adapts by freeze tolerance while some non-marine aquatic turtles are anoxia-tolerant (76). Mimicking the freeze tolerance and anoxia tolerance mechanisms could be advantageous to improving cryopreservation results.

Furthermore, a strain of Gibel Carp (*Carassius gibelio*) has been discovered to possess genetic resistance to overwintering starvation with less apoptosis (77), pointing to fact that certain genetic manipulations might confer resistance to tissue damage on cryopreserved subjects.

Cardiopulmonary resuscitation (CPR) combined with external defibrillation has restored life to many declared clinically dead from cardiac arrest (78) however, studies have suggested that delayed defibrillation is associated with lower survival rates (79, 80). This poses a serious challenge to cryonics and prolonging survival after delayed time to CPR is therefore critical in reanimating cryonics patients. In a related study using cats, brain activity was restored in a significant population after an hour of global cerebral ischemia and administration of norepinephrine/dopamine, heparin, insulin, and acidosis buffers (81). These agents are potentially applicable in increasing the chances of survival.

### Minimizing Reperfusion Injury

Reperfusion injury is a term used to describe damage to tissues incurred from inflammatory responses and oxidation by toxic free radicals when blood circulation returns following ischemia lasting more than approximately 20 min (82, 83). In order to prevent reperfusion injury, different interventions have been employed. For example, microcirculation remodeling (84), administration of alpha-tocopherol (vitamin E) (85) and cilostazol (86) have been found beneficial against reperfusion injury in the spinal cord of rats. Pretreatment of cryonics subjects with vitamin E could be of added advantage as it reduces blood clotting and lacks the risk of gastric bleeding associated with aspirin (9) and administration of iptakalim has been found to improve cerebral microcirculation in mice (87). At Alcor, drugs including excitotoxicity inhibitors, nitric oxide synthase inhibitors and Poly ADP-ribose polymerase inhibitors are administered against reperfusion injury with further resistance conferred on the brain by anesthesia (10).

## Conclusion

The ultimate expectation of animal cryopreservation and cryonics is that the subject will be revived after re-warming, removal of cryoprotectant, tissue repair, and cure. Whether or not this goal comes to fruition in the nearest future is widely dependent on the extent of research and scientific contributions. From this review, it is evident that scientific studies in cryonics and cryopreservation of animals are limited. The major challenges in cryonics have been identified as cost, ethical issues, cryodamage and revival with the most problematic being revival as its possibility is hanging on hope. Cryodamage can be overcome with the use of effective and safe cryoprotectants. At the same time, the cost of cryonics and ethical constrains might be lessened if there is proof of the concept of revival. Currently, no studies have attempted to cryopreserve and revive whole animals because of legal issues, pessimism surrounding the practice and shortage of cryonics expertise. Approval and funding of more pro-cryonics studies will lead to several serendipitous discoveries that could be advantageous to cryomedicine and cryobiology even though reanimation is not achieved.

## Author Contributions

All the authors contributed significantly to the writing of the manuscript. ME, GB, and SG: writing—original draft preparation. ME, YH, XL, JX, and ST: writing—review and editing. ST: supervision and approval.

## Funding

This research was supported by Central South University.

## Conflict of Interest

The authors declare that the research was conducted in the absence of any commercial or financial relationships that could be construed as a potential conflict of interest.

## Publisher's Note

All claims expressed in this article are solely those of the authors and do not necessarily represent those of their affiliated organizations, or those of the publisher, the editors and the reviewers. Any product that may be evaluated in this article, or claim that may be made by its manufacturer, is not guaranteed or endorsed by the publisher.
